# Use of a Highly Antioxidant Diet in the Regulation of Adipose Tissue Secretion in Patients after the BIB Procedure

**DOI:** 10.3390/foods10051108

**Published:** 2021-05-17

**Authors:** Edyta Balejko, Jerzy Balejko

**Affiliations:** Department of Commodity Science and Quality Assessment, Process Engineering and Fundamentals of Human Nutrition, Faculty of Food Science and Fisheries, West Pomeranian University of Technology, 70-310 Szczecin, Poland; jerzy.balejko@zut.edu.pl

**Keywords:** antioxidants, obesity, metabolic diseases, BIB (BioEnterics Intragastric Balloon), IL-1, IL-6, TNF-α, PAI-1, AGT, 8-isoprostane

## Abstract

Obesity is a global problem. The secretory activity of adipose tissue causes inflammation and disturbs metabolic parameters. Low-invasive bariatric procedures are an alternative to surgical treatment, especially in individuals who do not qualify for surgery or in whom conservative treatment does not bring the expected results. The diets designed for bariatric patients contained an increased proportion of bioflavonoids. The dietary components were carefully selected to provide anti-inflammatory effects. The experimental diets showed an antioxidant capacity (TEAC) of 433–969 µM TE/100 g or 100 mL, reducing ability (FRAP) of 13–58 µM TE/100 g or 100 mL, and total polyphenol content of 80–250 mg catechins/100 g or 100 mL. Lower levels of adipocytokines were obtained in the blood of patients following the diet. The results of the present study showed the participation of some adipocytokines in the regulation of energy homeostasis, lipid metabolism, glucose level, blood pressure and inflammation. Diet therapy should yield positive results in the long term, with the possibility of using immune modulation in personalized therapy for metabolic syndrome.

## 1. Introduction

According to the WHO, obesity is reaching epidemic proportions on a global scale. Based on an Irish study using a British Foresight Obesity model, it was predicted that by 2030, overweight and obesity will reach 89% of males and 85% of females, thus increasing the incidence of obesity-related diseases [1]. The high correlation between obesity and the incidence of metabolic syndrome results from the endocrine function of adipose tissue. Most adipokines exhibit a pro-inflammatory effect. The higher the degree of obesity, the more advanced the chronic inflammation with associated disturbances in the parameters of arterial hypertension, lipid and glucose metabolism, and insulin resistance [2,3]. Conservative methods in the treatment of overweight and obesity are sometimes ineffective and maintaining reduced body weight is an exceptional problem. Bariatrics was recognized as the most effective way of dealing with this condition [4,5]. The technique of endoscopy, the introduction of a balloon to the stomach, and BIB (BioEntericsIntragastricBalloon) is a minimally invasive form of overweight and grade I obesity treatment. The study aimed to modify a standard weight-loss diet that was saturated with bioflavonoids, to achieve both a reduction in body weight as well as chronic inflammation.

The diets recommended so far after bariatric procedures lack biologically active components. It was assumed that such enrichment of the diet might give it the characteristics of functional foods.

## 2. Materials and Methods

The study included patients of the Sonomed Medical Centre in Szczecin, which had been treated by the BIB method for six months in the years 2017–2020. Group I (20 females) applied a low-energy diet based on the recommendations of the University of Nevada School of Medicine. Group II (20 females) applied the authors’ original modifications of standard recommendations. The average age of the studied patients was 35 ± 5 years, while the average BMI was 31.1 ± 2.1 kg/m^2^.

In order to assess the correctness of the composition of the recommended original authors’ diet, qualitative determinations were conducted in nutritional rations. Water content was determined according to AOAC 2004, (926.06). Total nitrogen content was determined by the Kjeldahl method, according to AOAC 2004, (46-08, Nx5.75). Total fat content was determined by the Soxhlet method, according to AOAC 2004, (32.2). The fatty acid composition was determined according to AOAC 2004, (Aa 9-86). Moreover, to add an anti-inflammatory character to the diet, the proportion of vegetable and fruit cocktails was increased. It was recommended to drink up to two servings a day. Cocktail #1 contained spinach, kale, orange, parsley leaves, apple, and kiwi, with the addition of ground flaxseed. Cocktail #2 was a mixture of raspberries, forest fruits, pumpkin, and pomegranate juice. Cocktail #3 contained beetroot, ginger, apple, and orange. Sample #4 included the components of solid meals and contained—dairy products, avocado, linseed oil, fish, poultry in aspic, eggs, and bakery products. Antioxidant activities were determined in the samples by the TEAC method. The activity of reducing antioxidants was determined by the FRAP method [6], while the total polyphenol content determined according to [7].

All of the following tests and assays were conducted in duplicate, prior to commencing the therapy and the use of diet and after the completion of the observation period. Body composition measurements were carried out using an IOI 353 analyzer (which has a CEO 123 certificate and meets the requirements of the MDD Directive 93/42/EEC concerning medical devices). All patients had their blood sampled under laboratory conditions. The total CH, HDL, LDL, and TG values were determined by the colorimetric method and the fasting blood glucose levels by the colorimetric method with hexokinase. The total 8-isoprostane concentration was determined by immunoenzymatic ELISA assays, using kits with catalogue No. 516,351 by the Cayman Chemical Company, USA. The IL-1, IL-6, and TNF-α cytokine levels were determined by immunoenzymatic Sandwich ELISA assays, using monoclonal antibody-coated microplates (Mab) targeted against various respective epitopes. Kits by the company DRG Diagnostics International, Inc., USA (catalogue Nos EIA-4437, EIA-4640, EIA-4641). The method accuracy for the determinations of TNF-α concentration was ± 1% ± 0.010 absorbance, with a measurement range of 7–500 pg/mL. The method accuracy for the determinations of IL-1β concentration was ±1% ± 0.010 absorbance, with a measurement range of 0.35–1200 pg/mL. The method accuracy for the determinations of IL-6 concentration was ±1% ± 0.010 absorbance, with a measurement range of 2–2600 pg/mL. The angiotensinogen (AGT) and plasminogen PAI-1 levels were determined by immunoenzymatic ELISA assays, using kits with catalogue Nos. EO605h by the company EIAabScience Co Wuhan (catalogue Nos E0797h, E0532h).

Statistical analysis of the study results was conducted using the Statistica software versions 10, 12, and 13. The test power analysis was performed at α = 0.05. Since the calculated test power was 0.87, the likelihood of the occurrence of the effect was high.

In order to demonstrate the statistically significant differences between the groups, the non-parametric Wilcoxon signed-rank test was applied, with *p* assumed to be <0.05, for the results of the studied parameter concentrations, comparing the data before commencing the BIB therapy with the data after completing the BIB therapy.

## 3. Results

Biochemical analyses were carried out in the nutritional rations of the authors’ original diet. The diet included three main meals with average contents of the following: 80–85 g protein per day, 70 g complex carbohydrates per day, and 42 g total fat per day.

Moreover, the diet included two supplementary meals of either cocktails or mousses prepared mainly from vegetables with antioxidant properties. The meals and cocktails exhibited varying antioxidant properties (Table 1). The cocktails were richer in phenolic compounds than the meals, and their bioactive properties allowed them to be ranked in terms of the determined antioxidant capacity—cocktail #2 (with the highest total polyphenol content of 250.51 mg catechin/100 mL) > cocktail #3 (with a medium total polyphenol content of 159.49 mg catechin/100 mL) > cocktail #1 (with the lowest polyphenol content 120.51 mg catechin/100 mL). It was demonstrated that in cocktail #2, the greatest antioxidant effect was achieved by the addition of pomegranate juice. The total antioxidative status of pomegranate juice was determined to be 4.60 mmol/100 mL, and the total polyphenol content was 115 mg/100 mL. As for an orange fruit, it was demonstrated that the total antioxidant status was 2.17 mmol/100 mL, and the total polyphenol content was 53.4 mg/100 mL.

The reconstructed nutritional rations of patients contained 80 mg catechin/100 mg, i.e., they had the lowest antioxidant potential. It must be stressed that the cocktails might have modified the diet towards the anti-inflammatory effect.

The diets used in both groups following the BIB procedure resulted in reduced body weight. An average drop in body weight from 89.5 ± 4.4 kg to 72.2 ± 0.1 kg was achieved, which corresponded to a reduction in BMI from 31.1 ± 2.1 kg/m^2^ to 25.6 ± 2.3 kg/m^2^. A reduction in the adipose tissue weight was achieved, on average from 37.0 ± 5.1 kg to 25 ± 3.2 kg. A reduction in the visceral fat surface was demonstrated, in group I from 155.8 ± 16.2 cm^2^ to 125.5 ± 12 m^2^, while in group II from 149.5 ± 10.3 cm^2^ to 110.4 ± 11.2 cm^2^.

The average patient systolic pressure and diastolic pressure values, before and after the procedure are presented in Table 2. The optimal pressure values for an adult are considered to be 120 mm Hg (the so-called systolic pressure) and 80 mm Hg (the so-called diastolic pressure). Blood pressure is normal when the values do not exceed 120–129/80–84 mm Hg (Guidelines for the Management of Arterial Hypertension, 2019).

In accordance with the recommendations, the normal fasting blood glucose concentration values were assumed to be 70–99 mg/dL (3.4–5.5 mmol/L) [8]. Table 3 presents changes in blood glucose concentrations in patients after the diets used.

According to the standards of the European Society of Cardiology, the normal blood levels of total cholesterol (TCH) were 150–190 mg/dL, the LDL cholesterol fraction was less than 115 mg/dL, the HDL cholesterol fraction in females were more than 50 mg/dL, and the normal triglyceride levels ranged from 35 to 150 mg/dL (Table 4) (European Society of Cardiology, 2016).

The quantitative determination of isoprostanes provides a reliable and sensitive indicator of in vivo lipid peroxidation changes, thus, enabling the estimation of the contribution of free radicals to the pathophysiology of many diseases. Table 5 presents changes in isoprostane concentrations as an important part of the observation of plasma biochemical changes in group II patients.

The concentrations of selected cytokines, i.e., TNF-α, IL-1β, and IL-6, were determined by the ELISA method using the immunoenzymatic tests (Table 6). High levels of pro-inflammatory cytokines were noted in all patients, prior to commencing therapy. The results correlate positively with the excess adipose tissue, prior to the BIB procedure. After completion of the BIB procedure, a slight drop in the serum cytokine concentration was observed in group I patients, while in group II, a significant benefit was achieved. The effect was related to the satisfactory reduction in body weight and the visceral adipose tissue during the observation.

In the serum of patients from both groups, prior to balloon implementation and after the procedure, the levels of angiotensinogen (AGT) and plasminogen activator inhibitor-1 (PAI-1), i.e., proteins associated with cardiovascular function were determined (Table 7).

## 4. Discussion

In order to ensure therapy efficiency and long-term weight loss results, scientists emphasize the importance of comprehensive care for bariatric patients [9]. Obesity is a chronic inflammatory disease, as demonstrated by changes in the values of certain laboratory tests. A chronic inflammatory condition increases the risk of developing or exacerbating the symptoms in the course of arterial hypertension, diabetes mellitus, atherosclerosis, and metabolic syndrome. Based on the possibility of using functional ingredients in supporting the treatment of obesity, an attempt was made to formulate a diet with functional characteristics. The meals in the authors’ original diet were supplemented with cocktails with a high antioxidant potential. During observations, a reduction in body weight, including adipose tissue, was achieved in both groups. It was important to achieve a significantly more effective result in reducing the visceral adipose tissue in group II. Visceral tissue is more metabolically active than subcutaneous tissue [10]. It is distinguished by increased glucose uptake and the generation of fatty acids, which promotes insulin sensitivity and type 2 diabetes mellitus [11]. The weight and anatomical proximity of perivascular adipose tissue affect the quality of vascular endothelial cells, which explains the increase in cardiovascular problems among obese individuals [12]. The location of fat affects the functioning of organs and tissues [2,10]. As reported by Kassaian and Lu [13,14], the diversity of bioactive ingredients found naturally in the diet, including bioflavonoids and natural spices, e.g., cinnamon, rosemary, ginger, saffron, and turmeric, supports the loss of body weight. A study by Lu et al. analyzed the effect of the above-mentioned components on obesity and assessed molecular mechanisms in cellular, animal, and human models. Bioactive compounds from these spices restrict the accumulation of lipids in fat cells by regulating the expression of transcription factors. They might also modulate the activity of certain enzymes associated with lipogenesis, such as acetyl-CoA carboxylase (ACC), fatty acid synthase (FAS), glycerol-3-phosphate dehydrogenase (GPDH), and others. Following oral administration of spice extracts, intensified thermogenesis in the adipose tissue along with a reduction in body weight in obese mice and humans were demonstrated [14].

The effectiveness of bariatric procedures is assessed not only based on the reduction in the BMI value but also depending on the improvement of biochemical and morphological test results in obese patients. Most average biochemical test results were significantly different between the groups. Obesity is known to be accompanied by other metabolic diseases. The remission rate is a very important factor in assessing the effectiveness of bariatric surgical procedures. In the authors’ original study, a reduction in body weight, including (significantly) in the visceral adipose tissue following the application of a bioflavonoid-rich diet, had a significant impact on the regulation of glucose metabolism. In addition to energetic restriction, including the minimization of simple sugar proportion, the diet applied was saturated with bioactive ingredients exhibiting hypoglycemic effects. There are many reports confirming the effectiveness of bariatric surgery in the regulation of blood glucose regulation [15]. Hyperglycemia accompanying obesity consequently promotes systemic dyslipidemia while exhibiting a mutual relationship [16]. The authors’ original study of group I patients showed no significant, beneficial regulation of the lipid management fraction concentration. In group II, despite achieving no reduction in the total cholesterol levels to normal values, satisfactory results were achieved for the regulation of HDL/LDL and TG fractions and proportions. The effect of lipid metabolism, glycemia, and arterial blood pressure could be attributed to numerous factors. In addition to limiting the calorific value of the meals and sugars, it was the active compound from the added vegetables, fruit, and spices that were dominant. The cocktails were enriched with parsley leaves and ginger for their anti-inflammatory and antiemetic effects (ginger contains bioflavonoids and gingerol) [17]. In addition, ginger is known to exhibit hypoglycemic, hypolipemic, antioxidant, and anti-aggregative effects [17,18].

Obese people are exposed to greater oxidative stress [19]. Analysis of isoprostane levels is used to monitor the oxidative stress level and evaluate the risk factors or the efficacy of therapy in the course of certain diseases, such as atherosclerosis, insulin-dependent diabetes mellitus, hypercholesterolemia, and obesity [20,21,22,23]. In obese patients, chronic platelet activation as well as increased synthesis of proaggregating isoprostanes due to increased oxidative stress were observed [24]. The loss of body weight reduced the synthesis of prothrombotic isoprostanes. Certain diet components such as vitamins, plant pigments, bioflavonoids, tannins, fatty acids, and other compounds belonging to the phytamin group are bioactive compounds whose amount in the diet was intentionally increased. Phytamins are a group of physiologically active plant components with an effect similar to that of vitamins. Phytamins are classified as phenolic antioxidants (flavonoids, polyphenols), phytoestrogens (isoflavones, lignans), tri-compounds (sulfides, thiols, and isothiocyanates), and carotenoids [25]. The authors’ original study achieved a significant reduction in isoprostane levels, following weight loss and simultaneous long-term effects of the diet, with great antioxidant potential. A significant drop in isoprostane levels was observed in patients with inflammation-based diseases, who were provided with higher doses of anthocyanins originating from blueberries, strawberries, currants [26,27,28], and cherry juice [28] in their diet. The consumption of 250 g blueberries for six weeks significantly contributed to a reduction in plasma isoprostane concentration [29]. Higher intake of vegetables at the expense of red meat also contributed to a drop in plasma isoprostane levels in patients [30]. The daily intake of five rations of vegetables, including considerable amounts of cabbage, resulted in a persistently low isoprostane concentration, as compared to individuals whose diet was dominated by red meat [31]. The groups with the strongest antioxidant activity contained in bioflavonoids include the catechol (o-dihydroxy) group in the B-ring, which exhibits a high capacity to capture oxygen radicals, while the pyrogallol (trihydroxy) group in the B-ring gives the molecule a higher antioxidant activity. They bind free radicals, thus preventing the damaging effects of lipid peroxidation and oxidative modifications of DNA and proteins [32].

In adiposopathy, increased macrophage infiltration and an increase in cytokine or chemokine levels were observed. Approximately, 30% of the circulating IL-6 originates from adipose tissue. The IL-6 levels are correlated with high BMI values and visceral obesity [33]. Persistently elevated IL-6 levels are associated with the development of insulin resistance [33] and metabolic syndrome [34]. Numerous studies demonstrated a high correlation between a reduction in body weight and a drop in concentrations of IL-1, IL-6, and TNF-α in patients after bariatric surgery [35,36]. IL-1 is a proinflammatory cytokine in obesity with a persistently elevated plasma concentration [37] that is secreted to the blood and has systemic effects. A review paper, that examines the effect of diet ingredients on the modulation of cytokine activity, demonstrated that the supplementation of diabetic patients with tocopherol, resulted in reduced IL-1, IL-6, and TNF-α serum levels [38]. TNF-α is also found among the main inflammatory response mediators. Along with IL-6, TNF-α is considered to be a negative regulator of the insulin pathway. Pina et al. [39] assessed obese patients with metabolic syndrome by reducing the inflammatory condition parameters to achieve a satisfactory drop in TNF-α. Netto et al. studied the inflammatory condition and the coagulation parameters immediately after bariatric procedures, and comparatively, after six months. Statistically significant differences in the decrease in TNF-α concentration were demonstrated, as compared to the high values noted before surgery [40].

The authors’ original study demonstrated high values of IL-1β, IL-6, and TNF-α serum levels in patients from both groups prior to the BIB surgery. Cytokine serum levels dropped slightly in group I patients, while their serum levels decreased significantly in group II patients applying the authors’ original diet.

Arterial hypertension in 78% males and 65% females is linked directly to abdominal obesity [41]. The study determined the levels of angiotensinogen (AGT) and plasminogen activator inhibitor-1 (PAI-1), i.e., proteins associated with cardiovascular function. AGT enters the renin-angiotensin system (RAS), which plays an important role in the regulation of arterial blood pressure, affects the cardiovascular system, and regulates fluids. Metabolic changes accompanying obesity and insulin resistance increase AGT synthesis and secretion by adipocytes.

It was demonstrated that TNF-α activates AGT gene expression in adipocytes. The renin-angiotensin-aldosterone system (RAAS) might contribute to the development of insulin resistance. On the other hand, it is insulin resistance that is one of the factors of excessive RAAS activity. It was demonstrated that insulin stimulated AGT synthesis by adipocytes [41,42,43]. Plasminogen inhibitor type 1 is an acute-phase protein involved in fibrinolysis. It is synthesized by adipocytes, hepatocytes, endothelial cells, and fibroblasts and is then secreted into the blood. Its highest concentration is achieved in the morning hours, which is directly related to the peak incidence of cardiovascular incidents, at this particular time [44]. A high correlation was demonstrated between the PAI-1 concentration and the adipose tissue content. In obese patients, the tendencies towards prothrombotic susceptibility are significantly increased. The stimulants are pro-inflammatory adipocytokines IL-1 and TNF-α [45]. PAI-1 is involved in the pathogenesis of ischemic heart diseases and reinfarction [46]. Plomgard’s team confirmed the stimulation of PAI-1 synthesis by TNF-α, while excluding IL-6 [47]. The authors’ original study demonstrated a positive correlation for AGT and PAI levels with a drop in glucose levels and arterial pressure parameters, in patients receiving the modified diet.

At the end of the observation, statistically significantly lower levels of IL-1, IL-6, TNF-α, AGT, PAI-1, and isoprostanes were noted, which were dependent on the reduction in visceral adipose tissue in the studied group, as compared to the control group. Therefore, it could be assumed that the drop in IL-1, TNF-α levels had an effect on the reduction in AGT, PAI-1, and isoprostanes, which possibly resulted in their effect on restoring the fibrinolytic capacity of the patients’ vascular endothelium and on the cardiovascular function.

## 5. Summary

After the completed therapy period, in the group receiving the authors’ original diet, a reduction was noted in the values of oxidative parameters and inflammatory condition biomarkers as well as biochemical parameters, which correlated with the reduction in the BMI value and the amount of visceral adipose tissue. The obtained results of the authors’ original study confirmed the value of appropriate dietary care for patients after bariatric surgery. The diet with functional characteristics offers opportunities for prevention and effective care. Therefore, it could be appropriate to enrich weight-loss diets with the antioxidant components of functional foods.

The authors’ original study design was approved by the Research Ethics Board of the District Chamber of Physicians in Szczecin (OIL-Sz/Mf/KB/452/06/05/2015).

## Figures and Tables

**Table 1:** Antioxidative capacity (TEAC), reducing power (FRAP), and total polyphenols—content in cocktails and meals.

Sample	TEAC		FRAP		Total Polyphenols	
	Mean [µM TE/100 g or 100 mL]	SD	Mean [µM TE/100 g or 100 mL]	SD	Mean [mg Catechin/100 g or 100 mL]	SD
1	480.02	13.12	195.23	10.12	120.51	3.47
2	969.21	58.21	737.41	66.43	250.51	18.97
3	611.13	24.34	452.21	44.21	159.49	2.22
4	433.22	24.18	78.50	2.23	80.00	3.357

**Table 2:** Blood pressure values [mm/Hg] of patients on weight-loss diet before and after the BIB procedure.

	Group I—Receiving a Standard Diet		Group II—Receiving a Modified Diet	
	$\mathrm{before}\;\mathrm{BIB}\;\left(\bar{x}\pm SD\right)$	$\mathrm{after}\;\mathrm{BIB}\;\left(\bar{x}\pm SD\right)$	$\mathrm{before}\;\mathrm{BIB}\;\left(\bar{x}\pm SD\right)$	$\mathrm{after}\;\mathrm{BIB}\;\left(\bar{x}\pm SD\right)$
diastolic pressure [mm/Hg]	140 ± 10	137 ± 7	148 ± 12	129 ± 8
systolic pressure [mm/Hg]	90 ± 4	87 ± 2	93 ± 4	84 ± 3

**Table 3:** Mean blood glucose [mg/dL] of patients on weight-loss diets before and after the BIB procedure.

	Group I—Receiving a Standard Diet		Group II—Receiving a Modified Diet	
	$\mathrm{before}\;\mathrm{BIB}\;\left(\bar{x}\pm SD\right)$	$\mathrm{after}\;\mathrm{BIB}\;\left(\bar{x}\pm SD\right)$	$\mathrm{before}\;\mathrm{BIB}\;\left(\bar{x}\pm SD\right)$	$\mathrm{after}\;\mathrm{BIB}\;\left(\bar{x}\pm SD\right)$
Glucose [mg/dL]	123 ± 3	102 ± 6	120 ± 3	86 ± 3

**Table 4:** Changes in the blood lipid fraction values of patients on weight-loss diets before and after the BIB procedure.

	Group I—Receiving a Standard Diet		Group II—Receiving a Modified Diet	
	$\mathrm{before}\;\mathrm{BIB}\;\left(\bar{x}\pm SD\right)$	$\mathrm{after}\;\mathrm{BIB}\;\left(\bar{x}\pm SD\right)$	$\mathrm{before}\;\mathrm{BIB}\;\left(\bar{x}\pm SD\right)$	$\mathrm{after}\;\mathrm{BIB}\;\left(\bar{x}\pm SD\right)$
TCH [mg/dL]	240	219	215	195
HDL [mg/dL]	42	38	39	82
LDL [mg/dL]	199	180	170	90
TG [mg/dL]	90	75	135	52

**Table 5:** Mean values of blood isoprostane concentration [pg/mL] of patients on weight-loss diets before and after the BIB procedure.

	Group I—Receiving a Standard Diet		Group II—Receiving a Modified Diet	
	$\mathrm{before}\;\mathrm{BIB}\;\left(\bar{x}\pm SD\right)$	$\mathrm{after}\;\mathrm{BIB}\;\left(\bar{x}\pm SD\right)$	$\mathrm{before}\;\mathrm{BIB}\;\left(\bar{x}\pm SD\right)$	$\mathrm{after}\;\mathrm{BIB}\;\left(\bar{x}\pm SD\right)$
Isoprostanes [pg/mL]	134 ± 15	123 ± 6	143 ± 87	94 ± 12

**Table 6:** Mean values of pro-inflammatory cytokine concentration of patients on weight-loss diets before and after the BIB procedure.

	Group I—Receiving a Standard Diet		Group II—Receiving a Modified Diet	
	$\mathrm{before}\;\mathrm{BIB}\;\left(\bar{x}\pm SD\right)$	$\mathrm{after}\;\mathrm{BIB}\;\left(\bar{x}\pm SD\right)$	$\mathrm{before}\;\mathrm{BIB}\;\left(\bar{x}\pm SD\right)$	$\mathrm{after}\;\mathrm{BIB}\;\left(\bar{x}\pm SD\right)$
TNF-α [pg/mL]	11.78 ± 6.3	9.12 ± 4.2	12.66 ± 5.7	7.01 ± 2.2
IL-1β [pg/mL]	39.87 ± 7.3	36.25 ± 5.3	37.64 ± 3.6	24.33 ± 7.4
IL-6 [pg/mL]	17.22 ± 7.7	15.12 ± 6.3	24.32 ± 13.6	12.69 ± 5.7

**Table 7:** Mean AGT and PAI-1 concentration [ng/mL] of patients on weight-loss diets before and after the BIB procedure.

	Group I—Receiving a Standard Diet		Group II—Receiving a Modified Diet	
	$\mathrm{before}\;\mathrm{BIB}\;\left(\bar{x}\pm SD\right)$	$\mathrm{after}\;\mathrm{BIB}\;\left(\bar{x}\pm SD\right)$	$\mathrm{before}\;\mathrm{BIB}\;\left(\bar{x}\pm SD\right)$	$\mathrm{after}\;\mathrm{BIB}\;\left(\bar{x}\pm SD\right)$
AGT [ng/mL]	1346 ± 33	1134 ± 42	1266 ± 57	701 ± 22
PAI-1 [ng/mL]	36.52 ± 3.1	34.14 ± 5.3	32.66 ± 1.7	16.5 ± 3.4

## Data Availability

Department of Commodity Science and Quality Assessment, Process Engineering and Fundamentals of Human Nutrition, Faculty of Food Science and Fisheries, West Pomeranian University of Technology, 70-310 Szczecin, Poland.
